# Exhaled and nasal nitric oxide in laryngectomized patients

**DOI:** 10.1186/1471-2466-10-4

**Published:** 2010-01-28

**Authors:** Matthias F Kramer, Bernhard Olzowy, Annette Bihler, Dorothea de la Motte, Dennis Nowak, Rudolf A Jörres, Holger Dressel

**Affiliations:** 1 Department of Oto-Rhino-Laryngology, Head- and Neck Surgery, Ludwig-Maximilians-University, Munich, Germany; 2 Institute and Outpatient Clinic for Occupational, Social and Environmental Medicine, Ludwig-Maximilians-University, Munich, Germany

## Abstract

**Background:**

Nitric oxide (NO) shows differing concentrations in lower and upper airways. Patients after total laryngectomy are the only individuals, in whom a complete separation of upper and lower airways is guaranteed. Thus the objective of our study was to assess exhaled and nasal NO in these patients.

**Methods:**

Exhaled bronchial NO (FE_NO_) and nasal nitric oxide (nNO) were measured in patients after total laryngectomy (n = 14) and healthy controls (n = 24). To assess lung function we additionally performed spirometry. Co-factors possibly influencing NO, such as smoking, infections, and atopy were excluded.

**Results:**

There was a markedly (p < 0.001) lower FE_NO _in patients after total laryngectomy (median (range): 4 (1-22) ppb) compared to healthy controls 21 (9-41) ppb). In contrast, nNO was comparable between groups (1368 *versus *1380 in controls) but showed higher variability in subjects after laryngectomy.

**Conclusions:**

Our data suggest that either bronchial NO production in patients who underwent laryngectomy is very low, possibly due to alterations of the mucosa or oxidant production/inflammation, or that substantial contributions to FE_NO _arise from the larynx, pharynx and mouth, raising FE_NO _despite velum closure. The data fit to those indicating a substantial contribution to FE_NO _by the mouth in healthy subjects. The broader range of nNO values found in subjects after laryngectomy may indicate chronic alteration or oligo-symptomatic inflammation of nasal mucosa, as frequently found after total laryngectomy.

## Background

The fraction of exhaled bronchial nitric oxide (FE_NO_) has emerged as an important non-invasive marker of airway inflammation since its discovery in exhaled air by Gustafsson in 1991 [1]. The assessment of FE_NO _is increasingly used in clinical practice, especially for monitoring eosinophilic airway inflammation. Compared to bronchial NO, the measurement of nasal nitric oxide (nNO) has been methodologically and clinically less intensively studied. Since its first description in 1993, it has been proposed that the highest contribution to nNO originates from the paranasal sinuses [2]. nNO exceeds FE_NO _by factors of 10 to 100, depending on the sampling method used. As even slight contaminations by nasal NO greatly affect the level of exhaled NO, it is essential to exclude the upper airways when determining FE_NO_. In clinical practice this is done by closing the soft palatine/velum while subjects exhale against a flow resistance through the mouth.

In patients after total laryngectomy upper and lower airways are anatomically separated and thus they are ideally suited for separate assessments of NO in these two compartments. Currently there are no studies on both FE_NO _and nNO in these subjects. The measurements are of interest, since numerous factors can influence FE_NO_, such as respiratory tract inflammation, atopy, smoking, drugs, gender, age, and height [3]. There are data indicating a substantial contribution to FE_NO _arising from the mouth in healthy subjects [4]. On the other hand patients after laryngectomy show ultrastructural alterations of the lower respiratory tract - comparable to long-term tracheotomized, never-smoking patients [5] - likely due to the fact that they directly inhale ambient air. Same is reported for alterations within their nasal mucosa [6,7].

Thus the objective of our study was to assess exhaled and nasal NO in such patients with anatomically separated lower and upper airways. For comparison we included a group of healthy individuals.

## Methods

### Patients

The study was performed in cooperation between the Department of Oto-Rhino-Laryngology, Head and Neck surgery and the Institute and Outpatient Clinic for Occupational, Social and Environmental Medicine, both Ludwig-Maximilians-University, Munich, Germany. The study was approved by the institutional Ethic Committee, and subjects gave written informed consent. Specific exclusion criteria for this study comprised: atopy assessed by a screening test (see below), smoking, current respiratory tract infections, and anti-inflammatory drug intake assessed by questionnaire.

14 patients after total laryngectomy could be recruited. The median (range) time since laryngectomy had been performed was 6 (2-24) years before the study. Laryngectomy was done due to T3 or T4 squamous cell carcinoma of the larynx or hypopharynx. All patients underwent accelerated, adjuvant radiochemotherapy after surgery. By enrollment into the study all of them had at least 2 years of carcinoma-free survival and negative staging results, thus no signs of regional carcinoma, metastasis or second carcinoma such as bronchial cancer.

Patients were well adapted to their physical impairments arising from laryngectomy (e.g. permanent tracheotomy, impairment of speech, smell). They were asked concerning other diseases and symptoms by questionnaire. None of them reported a diagnosis or typical symptoms of asthma, atopic diseases, or nasal pathologies, e.g. polyposis or chronic rhinosinusitis. Before surgery all but one patient had been smokers with a median (range) of 22.75 (0-83) pack years. After surgery all of them refrained from smoking. As controls we measured 24 healthy, non-atopic, non-smoking individuals. For subjects' characteristics see table 1.

**Table 1:** subjects' characteristics

	Patients after total laryngectomy (n = 14)	Healthy controls (n = 24)
**Sex (m/w)**	12/2	13/11

**Age (years)**	64 (46-77)	37 (20-73) *

**Height (cm)**	174 (160-190)	171 (158-195)

**Weight (kg)**	75 (44-110)	69 (53-100)

**IVC (% predicted)**	97 (78-126)	103 (71-164)

**FEV_1 _(% predicted)**	98 (56-132)	106 (78-166) *

**FEV_1_/IVC (% predicted)**	104 (62-128)	106 (85-118)

Subjects' characteristics, displayed as absolute values or median (range).

IVC = maximal inspired vital capacity, FEV_1 _= forced expiratory volume in one second, * = p < 0.05

### Measurement of nitric oxide

Due to logistic and technical reasons, FE_NO _was determined in control subjects using a handheld electrochemical analyzer (NIOX mino, Aerocrine, Sweden), and in patients with total laryngectomy by a chemiluminescence analyzer NOA 280™ (Sievers, Boulder, Co, USA). These two types of analyzers have been shown to yield comparable results [8], and measurements were performed according to international guidelines [3]. There are however some data indicating a systematic difference between the two types of analyzers [9]. Thus we additionally compared the two devices in 15 subjects and found that geometric mean values were 27.1 and 26.4 ppb, respectively, with a Pearson correlation coefficient of 0.99. The mean difference between FE_NO _values obtained by both methods was 1.1 ppb, the 95% limits of agreement were -6.3 and 8.5 ppb. Thus we considered the devices to be comparable.

Subjects exhaled through a mouthpiece against a positive pressure of around 10 cm H_2_O, aiming to achieve a flow rate of 50 mL/s under visual control on a computer screen or the display of the handheld device. The analyzer was calibrated regularly using a certified calibration gas (Linde AG, Munich, Germany). Measurements were performed in triplicate. In patients with total laryngectomy FE_NO _was assessed by exhaling into the analyzer, while the tracheostoma was hermetically sealed by commercially available tracheotomy plaster, designed to be connected to speech prostheses (Provox^® ^Xtra-Base™, Atos Medical, Hörby, Sweden). When connecting the analyzer to the plaster we checked for a gas-tight fitting. Using this approach it was possible to measure easily and reproducibly FE_NO _in the patients.

Additionally nasal NO was determined in all patients after laryngectomy and in a subgroup of the healthy subjects (n = 11). Nasal NO was assessed by the chemiluminescence analyzer putting a nasal olive in one nostril, with the other nostril open and using a suction flow of 240 mL/min. In the control subjects velum closure was secured by slow oral expiration against a resistance that ensured a minimum pressure of 5 cmH_2_O. nNO was assessed when a plateau was reached. Measurements were performed in triplicate for each nostril and mean values of both nostrils were used for analysis. Velum closure was not necessary in patients after total laryngectomy due to surgical separation of upper and lower airways.

### Spirometry

After NO measurements spirometry was performed following established guidelines [10] using a pneumotachograph-based device (Masterscope™, Viasys Health Care GmbH, Höchberg, Germany). We recorded maximal inspired vital capacity (IVC), forced expiratory volume in one second (FEV_1_), and FEV_1_/IVC. Values were expressed as percent predicted using the European Respiratory Society reference values [11]. At least three technical acceptable flow-volume maneuvers were performed and the highest values were taken. In patients after total laryngectomy we connected the tracheotomy to the spirometer in the same way as described for FE_NO_.

### Atopy screening test

Atopy was excluded by history and/or a positive allergy screening test SX1 (Phadiatop^®^, Phadia, Freiburg, Germany). The sensitivity of this screening method is greater than 96% [12].

### Statistical analysis

As FE_NO _values are known to have a skewed distribution and due to the low number of subjects in the groups data are generally displayed as median (range). For nNO values, which were calculated as the mean of 6 single measurements (3 from each nostril) coefficients of variation are shown. For statistical comparisons between groups Fisher's exact test or Mann-Whitney-U-Test were used. P-values < 0.05 were considered statistically significant. Calculations were performed using SPSS 16.0 (SPSS Inc., Chicago, IL).

## Results

### Patients' characteristics

There was a significant difference concerning age (median age 64 versus 37 y; table 1) between patients after laryngectomy and healthy controls, otherwise both groups were comparable concerning gender, weight, and height. Additional factors possibly influencing NO, such as atopy, anti-inflammatory drugs, smoking, and infections had been excluded prior to enrolment in all subjects.

### Exhaled nitric oxide

**Figure 1:**
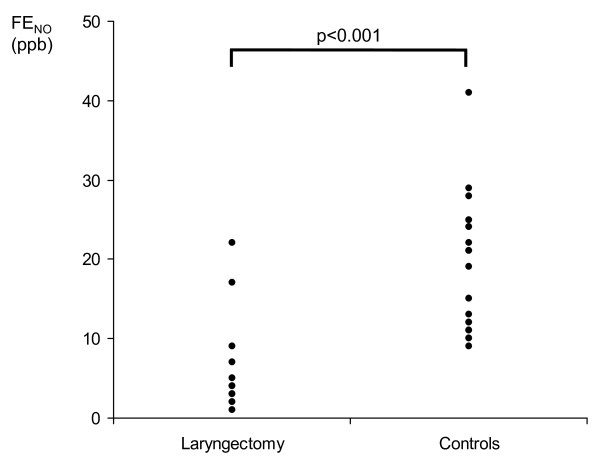
**Exhaled bronchial nitric oxide (FE_NO_) in patients after total laryngectomy and healthy control subjects**.

FE_NO _values in control subjects were within the normal range of healthy individuals with a median (range) of 21 (9-41) ppb. In patients after total laryngectomy median FE_NO _was 4 (1-22), and thus significantly (p < 0.001) lower (figure 1). In the patients only 2 subjects (14%) had FE_NO _values > 9 ppb, whereas in the controls only one subject (4%) had a value < 10 ppb.

### Nasal nitric oxide

**Figure 2:**
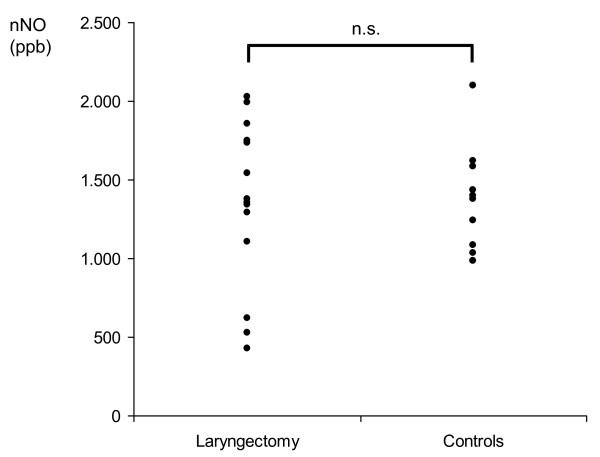
**Nasal nitric oxide (nNO) in patients after total laryngectomy and healthy control subjects**.

The median (range) coefficients of variation of the single 6-fold measurements of nNO were 11.1 (0.7-30.6) in the patients and 5.4 (0.8-25.2) in healthy subjects (n.s.). Nasal nitric oxide was 1368 (431-2028) ppb in patients after total laryngectomy and 1380 (988-2097) ppb in healthy controls (n.s.). As displayed in figure 2, the variability of nNO values was more pronounced in the patients, including some low values in the range of 500 ppb.

### Spirometry

IVC as well as FEV_1_/IVC in terms of %predicted values were comparable between groups, whereas FEV_1 _%pred was lower in patients after total laryngectomy (p < 0.05, table 1). Four patients (29%) had a FEV_1 _< 80%pred.

## Discussion

The present study demonstrated a significantly decreased level of FE_NO _in patients after laryngectomy compared to healthy control subjects, whereas nNO was similar between groups with a broader range of values in the subjects after laryngectomy. Total laryngectomy, with consecutive elimination of the climate function of the nose, might result in functional and structural changes of the lower airways, as described mainly within the first 6 months after surgery [13]. So far there are no data about the long-term effect this might have on exhaled NO. Our data suggest that bronchial NO production might be lowered in these patients, possibly due to alterations of the mucosa due to the exposure to inhaled dry ambient air that has not been humidified and filtered by the upper airways. However, two of the subjects after laryngectomy had FE_NO _values > 9 ppb (17 and 22 ppb) and thus in the range of the healthy controls. In those two patients laryngectomy had been performed 22 and 24 years before the study, the longest time periods of all participants. Eleven of the remaining 12 subjects had their operation within the last 12 years before the study and all of them had FE_NO _values below 10 ppb. The rise in FE_NO _only after a very long time since the laryngectomy had been performed may point to additional long term changes in the physiology of the mucosa.

On the other hand, the reduction of FE_NO _values might have been due to exclusion of other NO sources within the upper airways. There might be substantial contributions to exhaled NO originating from the larynx, pharynx and/or oral cavity. In line with this, mouth-washing reduced FE_NO _up to 50% [4], while intake of nitrate-rich food elevates FE_NO _[14]. Moreover, data obtained in trachetomized patients have indicated significant contributions from oral compared to tracheal sources [15,16]. We are not aware of studies in which laryngeal or pharyngeal contributions to FE_NO _have been directly determined. FE_NO _assessed in orally exhaled air is thought to be primarily of bronchial origin. This is based on the fact that bronchial NO much exceeds alveolar NO, and that contaminations by nasal NO are excluded by functional separation of lower airways and the nasal cavity. This is achieved by closing the velum when exhaling against a resistance, thereby preventing NO from nasal and paranasal sources from entry into the exhaled air. Our data might additionally indicate that the procedure of velum closure is not sufficient to assess bronchial NO without bias. However, these findings and hypothesis do not question the value of FE_NO _as a biomarker of eosinophilic bronchial inflammation, which has been shown in numerous studies [3]. As we did not include subjects with laryngectomy plus asthma, allergic rhinitis or atopy no further conclusions about the diagnostic value of FE_NO _in those patients can be drawn.

The comparison of the two groups has to deal with a number of factors that are known to influence FE_NO_. Among these are airway inflammation, smoking, steroid therapy, age, gender, and height [3]. While we tried to minimize their impact by the inclusion criteria, still some differences inevitably remained. In some respects a better match of patients and controls would have been preferable. Control subjects were younger than patients. Consequently we cannot exclude an effect of age on our results. An increase of FE_NO _with age has been found [17], while other studies did not reveal such a dependence [18]. Based on the available data, the effect of age would be very small and values in the group of patients should have been even elevated.

Smoking habits in the past differed between the two groups but at the time of the study none of the subjects smoked. Smoking is a risk factor for laryngeal cancer, and all but one of the carcinoma patients had smoked before surgery. This may have lead to alterations in lung function or chronic obstructive pulmonary disease (COPD). Indeed, FEV_1 _%pred was lower in patients after total laryngectomy, four patients having a FEV_1 _< 80% pred, possibly indicating COPD. None of the subjects received any respiratory medication. Notably, results concerning FE_NO _in COPD are conflicting and most authors found elevated FE_NO _values [19-21] Smoking is known to decrease FE_NO_, and FE_NO _has been reported to differ between never- and ex-smokers [22]. In contrast, a large population-based study did not find such a difference [17]. Moreover, it is known that in subjects quitting smoking FE_NO _increases to about the level measured in normal non-smokers within 1-8 weeks after cessation [23,24]. All of the patients after total laryngectomy had stopped smoking after surgery for at least 2 years. Thus, the pronounced difference in FE_NO _between our groups cannot be explained by the smoking status.

As increased levels of FE_NO _have been reported in lung cancer [25], we enrolled only patients with at least 2 years of carcinoma-free survival. All of them were well adapted to their impairment due to laryngectomy and there were no signs of recurrent carcinoma of any type. As a result of the inclusion criteria, which aimed at minimizing other possible influences on FE_NO_, we included only 14 patients after laryngectomy. As alternative therapeutic approaches for T3 or T4 carcinoma of the larynx or hypopharynx, such as modified surgical techniques and powered instruments such as LASER, are increasingly used, the number of total laryngectomy decreases. Survival is still poor (estimated 5-year survival rate <40%) despite numerous modifications over the last decades. We consider as strengths of our work the use of homogeneous, well defined groups, enabling the detection of differences in FE_NO _even in a small sample.

There is one study which has aimed at separating the lower and upper airways by measuring neurological patients with tracheotomy who are usually tracheotomized in order to prevent aspiration [15]. In these patients, even cannula with inflated cuffs cannot avoid silent aspiration, and consecutively pneumonia with an impact on FE_NO_. Three out of the 10 patients studied previously even used silver cannula without inflatable cuff. The only situation, in which lower and upper airways are guaranteed to be fully separated, is total laryngectomy. The data of the present study are in line with that of the previous study. A second study also found significanty lower FE_NO _values in 5 patients with tracheostomy when exhaling through the tracheostomy compared to exhaling through the mouth [16]. Future studies may additionally apply extended NO analysis for a better characterization of the NO exchange in those patients [26]. Hypothetically the diffusion rate of NO could be decreased due to thickness of the mucosa whereas the level of NO in the airway tissue might be normal. Another possible explanation for the low FE_NO _values in the patients may be a higher oxidative stress due to an inflamed epithelium of the bronchial mucosa.

Regarding nasal NO we observed a broader range of values in patients. Nasal function and anatomy are known to change after laryngectomy due to the lack of ventilation, and in CT scans of paranasal sinuses mucosal swelling is a regular finding [6,7,27,28]. Deniz et al. describe a hypersecretory phase with increased nasal mucosal clearance in the early period after laryngectomy and an atrophy of the nasal mucosa with decreased clearance later [6]. A second study also found increased nasal and bronchial mucociliary clearance in the first months after laryngectomy and a decrease 6 years after surgery [28]. Generally a variety of changes, including also subclincal, ongoing nasal inflammation may affect nNO and thus explain the larger variation compared to the healthy subjects. Furthermore the coefficients of variation were somewhat, but not statistically significant higher in the patients, indicating less precise measurements in those subjects. None of the subjects reported nasal pathologies, e.g. polyposis or chronic rhinosinusits. However, as we did not perform endoscopy or computed tomography during the current visit we cannot rule out completely nasal pathologies. Clinically silent polyposis or hypertrophy of turbinates thus might explain part of the variability of nNO found in laryngectomized patients [29]. Further studies with more subjects are warranted to correlate nNO with morphological changes after laryngectomy. To better characterize the inflammatory processes in the bronchial and nasal mucosa of patients with laryngectomy those studies should also include an assessment of neutrophilic inflammation, e.g. by measuring H_2_O_2 _or interleukins involved in the neutrophilic response in exhaled breath condensate. As additional clinical implication for patients after total laryngectomy, our findings indicate that it is well possible to measure exhaled and nasal NO and to perform spirometry in these patients using standard equipment.

## Conclusion

In conclusion, we studied patients after total laryngectomy as an *in vivo *model, in which a complete separation of lower and upper airways is guaranteed. Using strict definition of inclusion criteria in order to minimize factors influencing NO, we found markedly lower FE_NO _in patients after total laryngectomy compared to healthy controls. These data might indicate that the functional separation of lower and upper airways in healthy individuals by velum closure does not suffice to assess bronchial NO without bias and/or that bronchial nitric oxide would be lower than measured by the routine method, since NO originating from the larynx, pharynx and/or mouth would contribute to exhaled NO. However, substantial alterations of the bronchial mucosa as a consequence of total laryngectomy might be a further explanation.

## Competing interests

The authors declare that they have no competing interests.

## Authors' contributions

All authors read and approved the final manuscript. MK and HD: designed the study, analysed and interpret the data, and did the drafting of the manuscript. Further both supervised the study. BO, AB, and DM: were responsible for the recruitment of patients, conducted the measurements and gave substantial contribution to the conception of the study. DN: gave substantial contribution to the design of the study, interpretation of data, revised the manuscript critically. RJ: gave substantial contribution to the design of the study and interpretation of data. Furthermore he provided advice for physiological or statistical questions.

## Pre-publication history

The pre-publication history for this paper can be accessed here:

http://www.biomedcentral.com/1471-2466/10/4/prepub

## References

[B1] Gustafsson LE Leone AM Persson MG Wiklund NP Moncada S Endogenous nitric oxide is present in the exhaled air of rabbits, guinea pigs and humans Biochem Biophys Res Commun 1991 181 852 857 10.1016/0006-291X(91)91268-H 1721811

[B2] Alving K Weitzberg E Lundberg JM Increased amount of nitric oxide in exhaled air of asthmatics Eur Respir J 1993 6 1368 1370 7507065

[B3] American Thoracic Society Documents ATS/ERS recommendations for standardized procedures for the online and offline measurement of exhaled lower respiratory nitric oxide and nasal nitric oxide Am J Respir Crit Care Med 2005 171 912 930 10.1164/rccm.200406-710ST 15817806

[B4] Zetterquist W Pedroletti C Lundberg JO Alving K Salivary contribution to exhaled nitric oxide Eur Respir J 1999 13 327 333 10.1034/j.1399-3003.1999.13b18.x 10065676

[B5] Roessler F Grossenbacher R Walt H Effects of tracheostomy on human tracheobronchial mucosa: a scanning electron microscopic study Laryngoscope 1988 98 1261 1267 10.1288/00005537-198811000-00020 3185081

[B6] Deniz M Uslu C Ogredik EA Akduman D Gursan SO Nasal mucociliary clearance in total laryngectomized patients Eur Arch Otorhinolaryngol 2006 263 1099 1104 10.1007/s00405-006-0111-1 17086431

[B7] Fisher EW Lund VJ Rutman A The human nasal mucosa after deprivation of airflow: a study of laryngectomy patients Rhinology 1992 30 5 10 1579812

[B8] Alving K Janson C Nordvall SL Performance of a new hand-held device for exhaled nitric oxide measurement in adults and children Respir Res 2006 20 67 10.1186/1465-9921-7-67 PMC1462993 16626491

[B9] Pizzimenti S Bugiani M Piccioni P Heffler E Carosso A Guida G Rolla G Exhaled nitric oxide measurements: correction equation to compare hand-held device to stationary analyzer Respir Med 2008 102 1272 1275 10.1016/j.rmed.2008.04.006 18586480

[B10] American Thoracic Society Documents ATS/ERS standardization of lung function testing: standardization of spirometry Am J Respir Crit Care Med 2005 26 319 338

[B11] Quanjer PH Tammeling GJ Cotes JE Pedersen OF Peslin R Yernault JC Lung volumes and forced ventilartory flows. Report working party standardization of lung function tests, European Community for steel and coal. Official statement of the European Respiratory Society Eur Respir J (Suppl) 1993 16 5 40 8499054

[B12] Kohl C Debelic M In vitro screening for inhalant allergy with multi SX 1 RAST (Phadiatop) Allergy 1991 46 245 250 10.1111/j.1398-9995.1991.tb00581.x 1897685

[B13] Ackerstaff AH Hilgers FJ Sequelae of total laryngectomy with special reference to rehabilitation of the voice and the lower airways HNO 1997 45 97 104 10.1007/s001060050098 9173078

[B14] Olin AC Aldenbratt A Ekman A Ljungkvist G Jungersten L Alving K Toren K Increased nitric oxide in exhaled air after intake of a nitrate-rich meal Respir Med 2001 95 153 158 10.1053/rmed.2000.1010 11217912

[B15] Tornberg DC Marteus H Schedin U Alving K Lundberg JO Weitzberg E Nasal and oral contribution to inhaled and exhaled nitric oxide: a study in tracheotomized patients Eur Respir J 2002 19 859 864 10.1183/09031936.02.00273502 12030725

[B16] Schedin U Frostell C Persson MG Jakobsson J Andersson G Gustafsson LE Contribution from upper and lower airways to exhaled endogenous nitric oxide in humans Acta Anaesthesiol Scand 1995 39 327 332 10.1111/j.1399-6576.1995.tb04071.x 7793210

[B17] Olin AC Rosengren A Thelle DS Lissner L Bake B Toren K Height, age, and atopy are associated with fraction of exhaled nitric oxide in a large adult general population sample Chest 2006 130 1319 1325 10.1378/chest.130.5.1319 17099006

[B18] Olivieri M Talamini G Corradi M Perbellini L Mutti A Tantucci C Malerba M Reference values for exhaled nitric oxide (reveno) study Respir Res 2006 7 94 10.1186/1465-9921-7-94 16813647 PMC1534026

[B19] Delen FM Sippel JM Osborne ML Law S Thukkani N Holden WE Increased exhaled nitric oxide in chronic bronchitis: comparison with asthma and COPD Chest 2000 117 695 701 10.1378/chest.117.3.695 10712993

[B20] Ansarin K Chatkin JM Ferreira IM Gutierrez C Zamel N Chapman KR Exhaled nitric oxide in chronic obstructive pulmonary disease: relationship to pulmonary function Eur Respir J 2001 17 934 938 10.1183/09031936.01.17509340 11488329

[B21] Ferreira IM Hazari MS Gutierrez C Zamel N Chapman KR Exhaled nitric oxide and hydrogen peroxide in patients with chronic obstructive pulmonary disease: effects of inhaled beclomethasone Am J Respir Crit Care Med 2001 164 1012 1015 11587988 10.1164/ajrccm.164.6.2012139

[B22] Malinovschi A Janson C Holmkvist T Norbäck D Meriläinen P Högman M Effect of smoking on exhaled nitric oxide and flow-independent nitric oxide exchange parameters Eur Respir J 2006 28 339 345 10.1183/09031936.06.00113705 16641119

[B23] Robbins RA Millatamal T Lassi K Rennard S Daughton D Smoking cessation is associated with an increase in exhaled nitric oxide Chest 1997 112 313 318 10.1378/chest.112.2.313 9266863

[B24] Högman M Holmkvist T Walinder R Meriläinen P Ludviksdottir D Hakansson L Hedenström H Increased nitric oxide elimination from the airways after smoking cessation Clin Sci (Lond) 2002 103 15 19 10.1042/CS20020004 12095399

[B25] Masri FA Comhair SA Koeck T Xu W Janocha A Ghosh S Dweik RA Golish J Kinter M Stuehr DJ Erzurum SC Aulak KS Abnormalities in Nitric Oxide and Its Derivatives in Lung Cancer Am J Respir Crit Care Med 2005 172 597 605 10.1164/rccm.200411-1523OC 15947282 PMC2718532

[B26] George SC Hoegman M Permutt S Silkoff PE Modeling pulmonary nitric oxide exchange J Appl Physiol 2004 96 831 839 10.1152/japplphysiol.00950.2003 14766761

[B27] Calderazzi A Vaglini F Baschieri D Bruno R Donati F Sbragia-Daddi C Radiological changes in the nasal and paranasal cavities of patients having undergone laryngectomy Radiol Med (Torino) 1987 74 396 398 3685464

[B28] Maurizi M Paludetti G Almadori G Ottaviani F Todisco T Mucociliary clearance and mucosal surface characteristics before and after total laryngectomy Acta Otolaryngol 1986 102 136 145 10.3109/00016488609108658 3739687

[B29] Maniscalco M Sofia M Weitzberg E de Laurentiis G Stanziola A Rossillo V Lundberg JO Humming-induced release of nasal nitric oxide for assessment of sinus obstruction in allergic rhinitis: pilot study Eur J Clin Invest 2004 34 555 560 10.1111/j.1365-2362.2004.01384.x 15305890

